# Developing process measures in value-based healthcare: the case of aortic valve disease

**DOI:** 10.1136/bmjoq-2019-000716

**Published:** 2019-11-07

**Authors:** Berdel Akmaz, Nina Zipfel, Roland A Bal, Benno J W M Rensing, Edgar J Daeter, Paul B van der Nat

**Affiliations:** 1 Department of Value-Based Healthcare, Sint Antonius Hospital, Nieuwegein, The Netherlands; 2 Erasmus School of Health Policy and Management, Erasmus University Rotterdam, Rotterdam, The Netherlands; 3 Scientific Center for Quality of Healthcare (IQ Healthcare), Radboud university medical center, Radboud Institute for Health Sciences, Nijmegen, The Netherlands; 4 Department of Cardiology, Sint Antonius Hospital, Nieuwegein, The Netherlands; 5 Department of Cardiothoracic Surgery, Sint Antonius Hospital, Nieuwegein, The Netherlands

**Keywords:** value-based healthcare, aortic valve disease, surgical aortic valve replacement, transcatheter aortic valve replacement, process measures

## Abstract

**Background:**

As process measures can be means to change practices, this article presents process measures that impact on outcome measures for surgical aortic valve replacement (SAVR) and transcatheter aortic valve replacement (TAVR) within value-based healthcare.

**Methods:**

Desk research and observations of patient trajectories were performed to map the processes involved in TAVR and SAVR. Semistructured interviews were conducted with healthcare professionals (n=8) and patients (n=2) to explore which processes were most important in relation to a standard set of outcome measures that was already monitored. Additionally, open interviews (n=2) were held to prioritise results. A focus group was performed for validation of the formulated process measures. Numerical data for these measures was not collected.

**Results:**

Process maps of the full cycle of care of TAVR and SAVR treatments in theory and in practice were developed. 28 processes were found important by interview participants due to their expected impact on patient-relevant outcomes. Seven processes were prioritised to be most important and were formulated into 12 process measures for both TAVR and SAVR: ‘Number of times that deficient information provision to SAVR patients causes negative outcomes’, ‘Type of TAVR/SAVR prosthesis’, ‘Brand of TAVR prosthesis’, ‘Number of times the frailty score of a TAVR/SAVR patient >75 years is measured’, ‘Time between TAVR/SAVR surgery indication and surgery’, ‘Number of times that anticoagulants are stopped within 3 days before surgery’, ‘Time in hours between TAVR/SAVR surgery and permanent pacemaker implantation’ and ‘Percentage of standardised pain measurements’.

**Conclusion:**

This study proposes an addition of select process measures to standard sets of outcome measures to improve healthcare quality. It illustrates a clear method for identifying process measures with impact on health outcomes in the future.

## Introduction

Recently, there has been a shift towards patient-relevant outcome measures in the Netherlands, notably value-based healthcare (VBHC), which defines outcomes as the actual results of delivered care.^1 2^ The core goal of VBHC is to improve value for patients, defined as the health outcome achieved relative to costs.^3^ To measure value, causality chains leading to patient-relevant outcome measures have been developed.^1^ Moreover, the concept of care delivery value chains (CDVCs) in VBHC helps practitioners to understand, improve and integrate the activities related to a medical condition in the full cycle of care.^4^ However, in practice, hospitals struggle to find ways to improve outcomes. Process measures could play a role in solving this problem because processes are partial predictors of outcomes.^1 5^ Outcomes may be appropriate quality measures, but the link between processes and outcomes before quality measurement is performed should be regarded.^5 6^ After quality measurement, redirecting resources towards the processes that have the greatest effect on outcomes could help to improve quality of care in the most efficient way.^6^ Process measures comprise ‘whether what is now known to be “good” medical care has been applied’.^7^ They can be seen as handholds for practice change and are often based on work-as-imagined (WAI), which covers what managers, regulators and authorities believe what happens in practice. When developing process measures it is important to consider work-as-done (WAD) as it reflects what practitioners found to work best in practice.^8^

In the Netherlands, VBHC is most advanced in cardiology and cardiovascular surgery. Processes are not commonly measured in surgery, but studies showed that differences in processes can be associated with improved surgical outcomes.^9^ Previous studies identified infection-related and general process measures for all surgeries.^9 10^ The Dutch Health and Youth Care Inspectorate defined a process measure for pain measurement.^11^ The Dutch Association for Intensive Care has identified process measures specifically for the intensive care unit (ICU).^12^ Some studies identified process measures for cardiac surgery, that can be found through the National Quality Forum that included several process measures for all cardiac surgery in its database.^13 14^ Process measures and their relationship with outcomes have been studied in depth for procedures such as coronary artery bypass grafting.^6 9 14^ Some outcome measures have been identified for aortic valve disease (AVD), such as deep sternal wound infection.^15 16^ However, little research has been done on processes and their relationship with outcomes for surgical aortic valve replacement (SAVR), transcatheter aortic valve replacement (TAVR) and conservative treatment, the three treatments for AVD.^14 16–20^ There is no complete set of process measures regarding the full cycle of AVD care.^21^ One study formulated quality measures for mechanical and biological aortic valves based on guidelines.^18^ The Netherlands Heart Registry (NHR), which measures heart disease outcomes to improve quality and transparency in participating cardiac centres, makes that distinction, too, for SAVR treatment. The NHR has also identified process measures for TAVR treatment.^16 20^ Further, process measures have been identified concerning for example the proficiency of physicians performing TAVR.^17 19^

Overall, most process measures in the literature are formulated for (cardiac) surgeries in general or do not consider the full cycle of care of AVD. This article illustrates how process measures can be embedded in the concept of VBHC due to their impact on outcomes. It focusses on AVD and identifies patient-relevant process measures for SAVR and TAVR with potentially the highest impact on patient-relevant outcomes.

## Methods

### Study design

For this qualitative explorative case study, data and theoretical triangulation were applied to increase internal validity, by carrying out desk research, observations and semistructured interviews. The results of the data collection were discussed in a focus group. All data collection was carried out by the primary researcher, that is, the first author (BA). The first author was a researcher that was not part of the treatment team of the hospital and therefore no relationship existed with the treatment team during the participatory observations, the interviews and the focus group.

### Setting

The study was conducted in the cardiac centre of a Dutch teaching hospital. This single case was selected purposefully since the hospital monitored a standard set of TAVR and SAVR outcome measures from the NHR already, while it did not measure processes in the full cycle of care for AVD.^15 16 20 22^ Therefore, this case illustrates the possibly beneficial relation between process measures and outcomes. Conservative treatment for AVD was not included in this study since a standard set of outcome measures was not yet developed at the time of the current study.

### Interview and focus group participants

During the semistructured interviews, healthcare professionals (n=8), a TAVR patient (n=1) and a SAVR patient (n=1) were interviewed individually. Purposive sampling was used to select interview participants in order to engage each profession involved in the full cycle of AVD care and to select patients of both TAVR and SAVR treatment.^22^ The healthcare professionals were a cardiothoracic surgeon (n=1), cardiologist (n=1), anaesthesiologist (n=1), perfusionist (n=1), data manager for cardiothoracic surgery (n=1), nurse on the postoperative ward for TAVR surgery (n=1), nurse specialist on the postoperative wards for SAVR surgery (n=1) and nursing head of the preoperative nursing ward for SAVR surgery (n=1). The sample size was considered sufficient since data saturation was reached after eight interviews. Subsequently, the same cardiologist and another cardiothoracic surgeon were interviewed in a second round of interviews (n=2) to prioritise the important processes that were identified in the first round.

The focus group (n=11) was also selected through purposive sampling and consisted of a cardiothoracic surgeon (n=1), perfusionist (n=1), cardiothoracic nursing department head (n=1), data manager (n=1), senior advisor for the board of directors (n=1), care manager (n=1), fellow cardiologist (n=1), neurologist (n=1) and anaesthesiologists (n=3). The sample size was deemed sufficient because all professions were represented. Notes taken during the focus group were transcribed and analysed.

### Data collection and analysis

Desk research focused on WAI^8^ and involved studying healthcare policies, protocols and patient brochures. In addition, CDVCs were readily available at the hospital to identify large parts of the processes and to prepare ‘theoretical’ process maps. The theoretical process maps followed five phases of the CDVC: ‘Diagnosing’, ‘Preparing’, ‘Intervening’, ‘Recovering and rehabbing’ and ‘Monitoring and managing’. ‘Monitoring and preventing’ was excluded from the process maps because this phase concerns a period before hospital treatment and takes longer time, such as early age dietary habits. Moreover, this phase differs for each patient; some are referred by other hospitals and others present at the outpatient clinic with new heart problems.

Participatory observations of patient trajectories took place with patients preoperatively (n=2), during surgery (n=4) and postoperatively (n=2). During the observations, informal interviews addressing questions about WAD^8^ took place, which added depth to the data. Field notes taken during the observations were transcribed and analysed. Subsequently, the theoretical process maps were revised and ‘practical’ process maps were developed.

Following, semistructured interviews with healthcare professionals and patients were conducted by the primary researcher. The aim was to investigate which processes were considered most important regarding their impact on patient-relevant outcomes. Patient interviews were performed to also elicit patient’s perspectives on that matter. Interview questions (online supplementary file I) were based on the CDVC and the WAD process maps. The standard set of outcome measures for TAVR and SAVR of the NHR that was already monitored in this hospital was used as a reference tool in the interviews to identify processes that could influence these outcomes (online supplementary file II).^15 16 20^ The WAD process maps were an additional interview tool during interviews with professionals to show them the full cycle of care of AVD and help them point out the processes that influence outcomes. The interviews were audio-recorded with consent of the participants. One participant did not give permission to record the interview. Instead, the interviewer took extensive field notes that were checked by the participant. To increase internal validity, the transcripts of the remaining interviews were sent to the participants for a member check.

10.1136/bmjoq-2019-000716.supp1 Supplementary data

The interviews were initially transcribed and analysed by the primary researcher, using ATLAS.ti V.8.0 software. Interview coding followed grounded theory, producing an overview of primary, secondary and tertiary codes.^22^ First, inductive content analysis took place with open coding. Then, axial coding deductively led to categories from the various labels. With selective coding, the five phases of the care cycle defined in the CDVC were used as categories for the axial coding terms. Each category was further divided into ‘Important processes’, ‘Improvements’ and ‘Improvements process map’, separately for TAVR and SAVR. The final category concerned improvements regarding the process maps. In order to ensure internal reliability, co-authors were given insight into coding work and codes were discussed among co-authors. Issues were resolved until consent was reached. Moreover, co-authors evaluated the results that were presented by the primary researcher following the analyses, to increase trustworthiness of results.

After the results of the semistructured interviews (n=8), a cardiologist and cardiothoracic surgeon were interviewed in a second round of interviews. These interviews aimed to prioritise the identified important processes from the first round of interviews and were used to define which processes were most important to translate into process measures. The interviews were open and began with the question: *Which processes in this list should be monitored as process measures in the future, considering their impact on outcomes?* Since the interviews were semistructured and open, the researcher was able to ask questions until depth was reached to increase internal validity.

Processes were defined important based on the number of times the measure was mentioned and the subsequent prioritisation by the cardiologist and the cardiothoracic surgeon. Subsequently, they were formulated into process measures by the primary researcher and were discussed in a focus group for validation. The primary researcher led the focus group, posing questions on how accurate the group members found the process measures and whether these could be improved. Numerical data for these measures was not collected since the purpose of this study was to illustrate how process measures can be embedded in VBHC due to their impact on outcomes.

To reach external reliability during data analysis, an audit trail was created by keeping a logbook about inconsistencies in results, which were resolved based on consent among the authors. Moreover, potential inconsistencies in results also came to light during the prioritisation interviews with the cardiologist and cardiothoracic surgeon.

### Patient and public involvement

Two semistructured patient interviews were performed and patients were included using purposive sampling. The outcome measures applied in this study are derived from the NHR.^16^ Patients were involved as part of a selection team for the development of these outcome measures.^23^

## Results

Theoretical process maps of how TAVR and SAVR treatments are ‘imagined’ were developed through desk research (online supplementary file III). Looking at how work is done in practice provided varying or additional descriptions of the processes taking place in the full cycle of AVD care. The practical process maps are shown in online supplementary file IV.

Interview participants found in total 28 processes within the full cycle of care of TAVR and SAVR important due to their impact on patient-relevant outcomes. After prioritisation by the cardiologist and the cardiothoracic surgeon, seven processes regarding TAVR and/or SAVR were identified as most important out of the 28 processes:

1. Information provision to patients about SAVR treatment.
2. Valve choice for TAVR and SAVR treatment.
3. Frailty screening of patients undergoing TAVR and SAVR treatment.
4. Managing waiting lists for TAVR and SAVR treatment.
5. Stopping anticoagulants in SAVR treatment.
6. Permanent pacemaker implantations in TAVR and SAVR treatment.
7. Pain measurement in patients after SAVR treatment.

The seven prioritised processes are elicited in the next sections. As can be seen, not all processes are important or applicable for both TAVR and SAVR. Moreover, three measures were formulated for ‘Valve choice’. Therefore, 12 process measures were formulated in total for both TAVR and SAVR as shown in table 1.^20^

**Table 1:** Process measures derived from the processes identified as most important

Process	Process measure	Treatment	Outcome measure	n*
1. Information provision to patients about SAVR treatment	1. ‘Number of times that deficient information provision to SAVR patients causes negative outcomes’	SAVR	Deep sternal wound infection	4
2. Valve choice for TAVR and SAVR treatment	2. ‘Type of SAVR prosthesis: bioprosthesis type unknown; stentless bioprosthesis; stented bioprosthesis; mechanical; homograft; autograft; adhesion-free bioprosthesis and unknown’ (NHR)^20^	SAVR	Valve re-intervention	2
	3.‘Type of TAVR prosthesis: balloon expandable; self-expandable and unknown’ (NHR)^20^ 4.‘Brand of TAVR prosthesis’	TAVR	Permanent pacemaker implantation	3
3. Frailty screening of patients undergoing TAVR and SAVR treatment	5.‘Number of times the frailty score of a TAVR patient >75 years is measured’	TAVR	Mortality	3
	6.‘Number of times the frailty score of a SAVR patient >75 years is measured’	SAVR		
4. Managing waiting lists for TAVR and SAVR treatment	7. ‘Time between TAVR surgery indication and surgery’	TAVR	Mortality	1
	8. ‘Time between SAVR surgery indication and surgery’	SAVR	Quality of life	2
5. Stopping anticoagulants in SAVR treatment	9. ‘Number of times that anticoagulants are stopped within 3 days before surgery’ (negative)	SAVR	Re-sternotomy → deep sternal wound infection	2
6. Permanent pacemaker implantations in TAVR and SAVR treatment	10. ‘Time in hours between SAVR surgery and permanent pacemaker implantation’	SAVR	Infection	2
	11. ‘Time in hours between TAVR surgery and permanent pacemaker implantation’	TAVR	Mobilisation → quality of life Infection	1
7. Pain measurement in patients after SAVR treatment	12. ‘Percentage of standardised pain measurements’^11^	SAVR	Lung infections Mobilisation → quality of life	2

*n=number of times the measure was mentioned by interview participants. n<3 signifies that the process measure was selected according to the prioritisation by the cardiologist or cardiothoracic surgeon.

SAVR, surgical aortic valve replacement; TAVR, transcatheter aortic valve replacement.

### Information provision to patients about SAVR treatment

Information provision about SAVR treatment is part of the standard care process. One participant thought that uncertainty, because of deficient (incomplete or confusing) information could lead to patients not knowing when to mobilise postoperatively, which could lead to sternal dehiscence, making otherwise preventable infections more likely. Thus, ‘information provision’ was suggested as a process measure for the outcome ‘deep sternal wound infection’.

### Valve choice for TAVR and SAVR treatment

The valve choice for TAVR patients depends on the size and access route (transfemoral or transapical) of the stent. Different suppliers produce different types and brands of TAVR stents. Participants mentioned valve choice for both TAVR and SAVR as important due to heart rhythm disturbances that can lead to the implantation of a permanent pacemaker:

​Heart arrhythmia has to do with the type of valve, because you have different types. One valve is placed a bit lower down and it can disturb the heart rhythm more than others do. This also applies to the TAVRs. (cardiothoracic surgeon)

However, SAVR valve choice cannot account for heart rhythm disturbances. The valve choice depends on the patients’ age and need for anticoagulant therapy: older patients (>65 years) are offered biological valves because these last 15 years. Anticoagulant therapy is not necessary with biological valves which is an advantage for both older and younger patients. According to the participants, the SAVR valve choice influences the outcome ‘valve re-intervention’. Valve re-intervention is also influenced by infections such as endocarditis. Moreover, valve choice is also influenced by gender: women who anticipate becoming pregnant receive biological valves to prevent bleeding during childbirth due to anticoagulation use after a mechanical valve.

To sum up, ‘valve choice for TAVR and SAVR’ could be a process measure for the outcome measure ‘permanent pacemaker implantation’ for TAVR and ‘valve re-intervention’ for SAVR. Correction for gender and age would be necessary when measuring SAVR valve choice in practice.

### Frailty screening of patients undergoing TAVR and SAVR treatment

Participants argued that it is important to distinguish when patients are too frail to be treated, especially TAVR patients who constitute an older and therefore vulnerable patient population. Being too frail is a contraindication for TAVR. This decision could impact mortality because it can lead to a shift in mortality rates: if surgery is done there is a probability that the patient might be deceased shortly after surgery due to frailty or live longer because of the treatment. If no intervention is carried out, 30-day mortality may be lower but, for example, more people could die in 1 year because they were not treated. Overall, a process measure for both TAVR and SAVR patients could be ‘measuring the frailty score’, which influences the outcome measure ‘mortality’.

### Managing waiting lists for TAVR and SAVR treatment

Both TAVR and SAVR treatments have waiting lists until intervention. After the decision for surgery, a long waiting list is unfavourable for TAVR patients because time-related complications can occur. The interviewed TAVR patient in this hospital had to wait longer than he/she had been led to expect. In turn, when a SAVR waiting list is too short, important tests could be missing. This can cause changes in surgery planning and lead to procedural delays, which could lower the quality of life. The interviewed SAVR patient pointed out that their waiting time was quite short. Thus, ‘waiting time’ was mentioned as a process measure for ‘mortality’ of TAVR patients and ‘quality of life’ of SAVR patients, where a balance in the length of the waiting list needs to be found.

### Stopping anticoagulants in SAVR treatment

When the patient is admitted to the ward, medication policy is different for TAVR and SAVR patients. TAVR patients need to receive platelet inhibitors before surgery and SAVR patients taking anticoagulants need to stop three days before surgery. Stopping anticoagulants on time is considered important because it can prevent re-sternotomy, which can be related to infections:

​Also important is stopping anticoagulants before surgery. People often get various anticoagulant drugs which do not affect valve re-intervention, but for example, do affect re-sternotomy, which is not in the table. But re-sternotomy is indirectly related to deep wound infection, so if you can reduce that one …’ (cardiothoracic surgeon)

Moreover, stopping anticoagulants on time influences the risk of bleeding and blood transfusions. ‘The number of times that anticoagulants were stopped within 3 days before surgery’, was mentioned as a negative process for the outcome measure ‘deep sternal wound infections’.

### Permanent pacemaker implantations in TAVR and SAVR treatment

All SAVR patients receive a temporary pacemaker. SAVR patients could risk having the temporary pacemaker leads in place for too long which can cause infections and bleeding:

How often do you actually still need them [pacemaker leads] and does that weigh against the fact that they are still in there? Letting them stay in there can cause infection and bleeding. (nursing head)

TAVR patients might receive a transvenous temporary pacemaker with which they are not allowed to move. If the temporary pacemaker can be removed or replaced by a permanent pacemaker quicker, there is a lower chance of infection and unnecessary bedridden time. Mobilisation can also start sooner and therefore quality of life improves:

​I think we need to remove everything faster. (…) That is certainly vital for old people. Out of bed quickly, everything out fast, all lines out, standing beside the bed quickly, yes. [Keep it in] as short [a time] as possible, the pacemaker. (cardiologist)

However, a temporary pacemaker should not be removed too quickly because a disturbed heart rhythm can also restore itself and prevent a permanent pacemaker:

​On the one hand I think it could be faster, if it is clear that someone needs it, then it should be done fast. But yes, that period until it is clear that it is necessary should not be too short either. So, say you wait two weeks to see if the rhythm gets better, then it is also fine to say after two weeks that a pacemaker is needed. (nurse specialist)

The TAVR and SAVR patients differed in this matter. The TAVR patient had to stay in bed for 5 days but wanted to mobilise quicker. However, the SAVR patient had already mobilised quickly in the ICU.

In sum, the ‘time until a permanent pacemaker’ was identified as a process measure for the outcome measures ‘infection’ (TAVR and SAVR) and ‘quality of life’ (TAVR). It remains a matter of discussion what would be an appropriate time for this measure.

### Pain measurement in patients after SAVR treatment

Postoperative pain monitoring after SAVR and TAVR surgery is considered vital. Pain management together with physiotherapy helps SAVR patients to breathe properly, which prevents lung infections. Pain scores must continue to be measured consistently:

Pain score is also important because if people are in pain and unconsciously inhale less deeply, then they risk getting atelectasis and then pneumonia. It is really important to measure the VAS score (patients can score the pain they feel from zero to ten on the Visual Analogue Scale (VAS)) so that they do not have any pain. (cardiothoracic surgeon)

Pain medication is important for mobilising the patient and having a pain team at a hospital is favourable. Both TAVR and SAVR patients pointed out that their pain was continuously measured.

Overall, ‘measuring pain scores’ could be a process measure that influences the outcome ‘lung infections’. In addition, ‘administration of pain medication’ may be a process measure for mobilisation, which could influence ‘quality of life’.

## Discussion

Our study identified an extensive list of process measures with highest impact on outcomes, covering all the phases of the full cycle of (AVD) care except for ‘Monitoring and preventing’. In this case study it appeared challenging in practice to achieve the ambition of VBHC of only measuring outcomes to improve quality of care. Our hypothesis is that solely focusing on outcome measures without taking their context into account, could lead to uncertainty about what is causing the unfavourable outcomes and where improvement is needed. Though, simply focusing on process measures without looking at the consequences for relevant outcomes could lead to improving the wrong aspects. Process measures are actionable and offer feedback about which quality improvement activities are needed to improve patient outcomes.^9 24^ They can often be measured more easily and quickly than outcomes. For example, data collection can be fed back continuously and in real time. In contrast, outcomes such as ‘quality of life’ may require extensive follow-up time.^1 24^ Therefore, it is recommended to focus on both types of measures. Using process measures in combination with outcome measurement should not be about guideline adherence, but about how processes influence outcomes and in what way outcomes can be improved through process optimisation.^1^ Standard sets of outcome measures can be defined and used for benchmarking, but the process measures that impact outcomes can differ between organisations and should not be included in obligatory registries.

This study clearly illustrates how processes could influence outcomes in VBHC. Whether using the identified process measures will influence and improve outcomes in practice requires further research. Further research is also recommended to develop process measures for multiple settings, besides AVD. The process measures in our study are considered a valuable addition to the existing process measures in the literature. The definitions of The Dutch Health and Youth Care Inspectorate and the NHR have been used for our pain management and TAVR and SAVR prosthesis type process measures.^11 16 20^ A substantiation for our process measures in the literature can be found in online supplementary file V.

Within the VBHC concept an outcome measure hierarchy to guide the development of outcome measures was proposed.^1^ However, there is no practical tool for developing process measures with impact on outcomes. This study drafts a proposal for a method to identify process measures. First, it recommends identifying the full cycle of care for a disease using the CDVC concept. Second, it is important to take differences between WAI and WAD into account when identifying processes. If the understanding of WAD is incomplete or incorrect, then the idea of a particular intervention (process measure) with a particular consequence (outcome measure) could fail.^8^ Our study supports this argument because new process maps after the observations (WAD) enhanced the reflection of the real-life situation. Third, interview results need to be validated by a focus group to confirm whether healthcare professionals agree with the definitions of measures to avoid ambiguity.^7^ A group needs to work together to formulate and measure the process measures, and therefore process measurement fosters teamwork.^1^ As in this study, it may take time or need further research to decide on definitions, such as how soon a permanent pacemaker implantation should take place. Finally, it is important to consider the feasibility of measuring the selected process measures. The processes should be discrete data that are recorded in for instance the electronic patient record, so that information can automatically be generated.^24^

### Limitations

While this case study was a good illustration of the possible relation between processes and outcome measures, performing this research at one single institution might limit the generalisability of the results. Though, process measures are also determined locally and are hospital-specific. Moreover, ‘Monitoring and preventing’ is important when considering the full cycle of care. However, the aim was to consider process measures that can be influenced within the hospital of this study and therefore this phase was beyond the scope of this study. Additionally, only two patients were interviewed. Yet, the goal was not to reach data saturation because after the interviews it became clear that patients have relatively little (technical) insight about which processes are important regarding their impact on expected outcomes. Furthermore, the same cardiologist from the first round of interviews was interviewed again in the second round to elicit his view on the priority of the processes, which might have influenced the results for prioritisation. Finally, unfortunately no cardiologist was available to participate in the focus group while this may have been an important additional view on the process measures.

## Conclusion

This study proposes working with a selection of process measures in addition to a standard set of outcomes to improve quality of care. Our study illustrates how process measures might be used to improve outcomes in VBHC. Besides case-specific process measures, we were able to identify a clear method for the identification of process measures with impact on health outcomes in the future.
