# Correction: Developing process measures in value-based healthcare: the case of aortic valve disease

**DOI:** 10.1136/bmjoq-2019-000716corr1

**Published:** 2019-12-06

Akmaz B, Zipfel N, Bal RA, *et al.* Developing process measures in value-based healthcare: the case of aortic valve disease. *BMJ Open Quality* 2019;8:e000716. doi: 10.1136/bmjoq-2019-000716.

The license type of the paper has changed from CC-BY-NC to CC BY.

