# Supplementary material for: Developing process measures in value-based healthcare: the case of aortic valve disease
Source: BMJ Open Qual. 2019 Nov 7;8(4):e000716. doi: 10.1136/bmjoq-2019-000716 (PMC6863668; doi:10.1136/bmjoq-2019-000716)
Supplement: Supplementary data [file bmjoq-2019-000716supp001.pdf]

Supplementary File I: Interview questions

1. Which processes regarding Diagnosing/Preparing/Intervening/Recovering and rehabbing/Monitoring and managing do you think are the most important because they could influence outcomes? And why?
2. How would you prioritise them based on important to unimportant?
3. Are there any improvements possible within these processes? How could one handle these processes the best?
4. Are any other complications possible which may impact outcomes that severe, that you would want to measure and track them?

Akmaz B, *et al. BMJ Open Qual* 2019; 8:e000716. doi: 10.1136/bmjoq-2019-000716

Supplementary File II: Outcome measures set of the NHR

Hierarchy	Generic outcome measures	SAVR-specific outcome measures	TAVR-specific outcome measures
**Survival**	120-day mortality<br>Long-term survival		Procedural mortality<br>30-day mortality
**Degree of recovery/ health**	Quality of life		NYHA classification
**Damage of the treatment (side effects, complications or medical mistakes)**		CVA<br>Deep sternal wound infection<br>Implantation new permanent pacemaker	CVA<br>Implantation new permanent pacemaker<br>Vascular complications
**Durability of recovery or health**		Freedom of valve re-intervention	Freedom of valve re-intervention

1-3

Akmaz B, *et al. BMJ Open Qual* 2019; 8:e000716. doi: 10.1136/bmjoq-2019-000716

Supplementary File III: Work-as-imagined TAVR and SAVR process maps

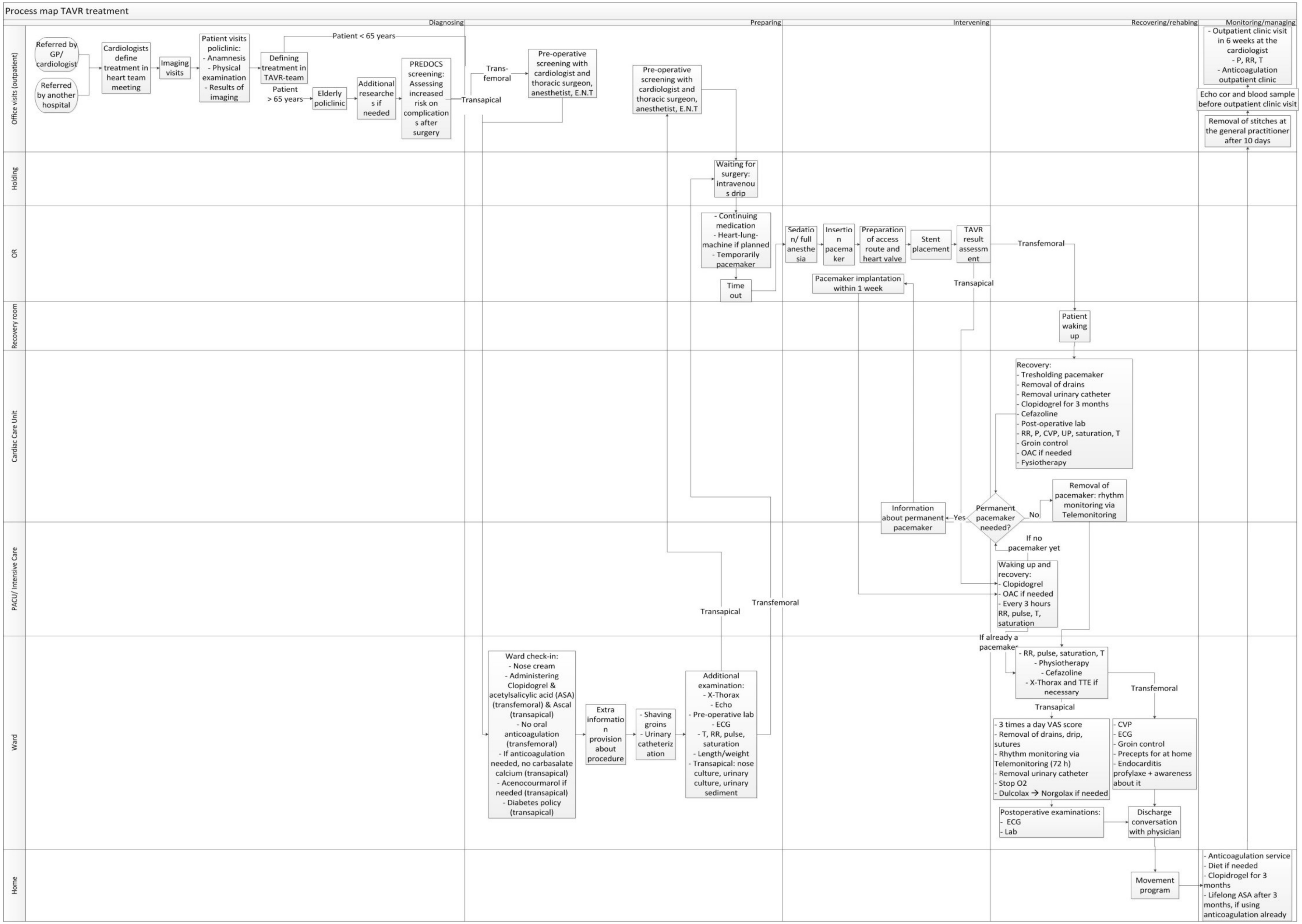

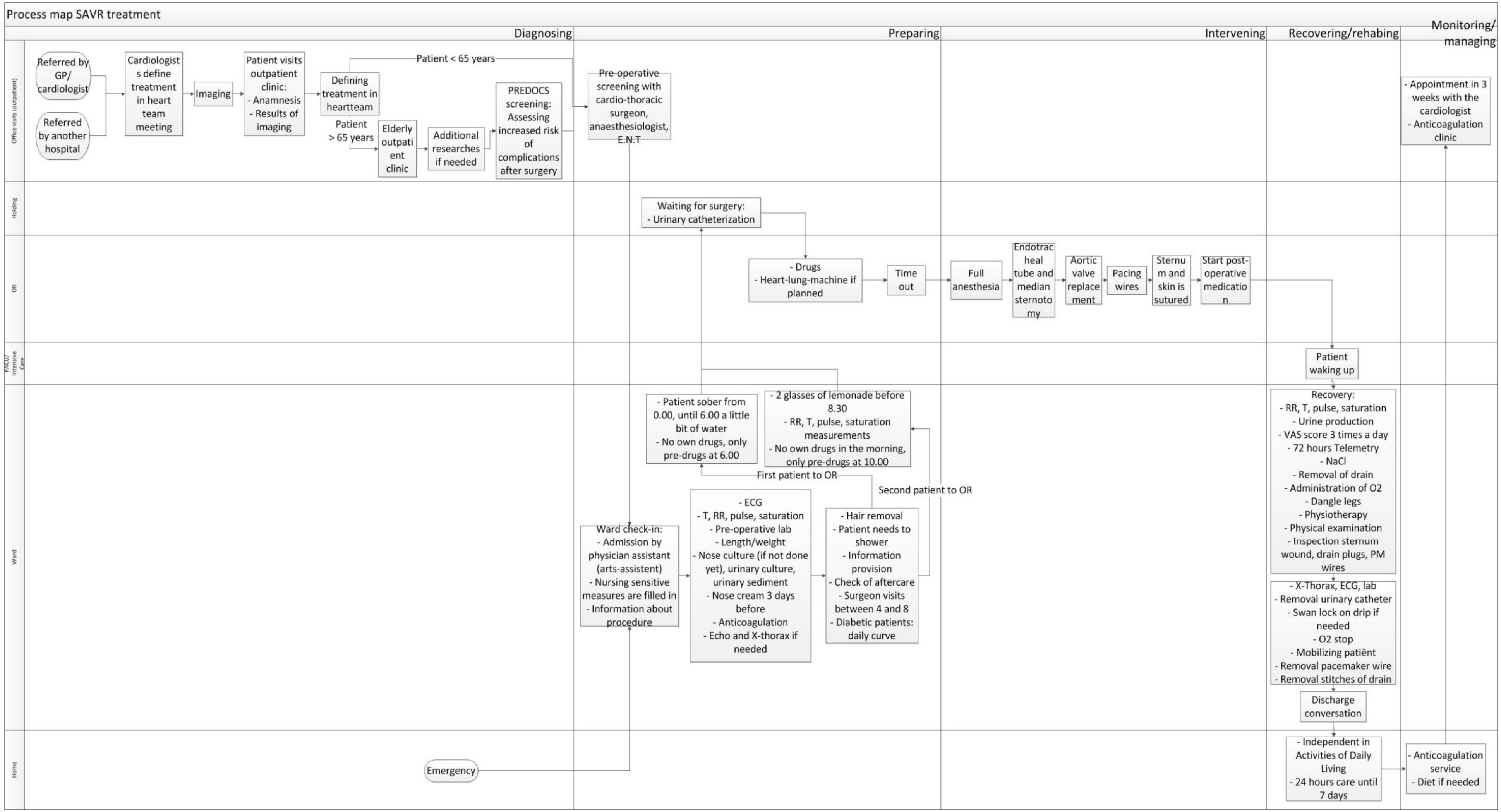
Process map SAVR treatment
Diagnosing
Preparing
Intervening
Recovering/rehabing
Monitoring/ managing
Office visits (outpatient)
Holding
OR
PACU/ Intensive Care
Ward
Home
Referred by GP/ cardiologist
Referred by another hospital
Cardiologists define treatment in heart team meeting
Imaging
Patient visits outpatient clinic: - Anamnesis - Results of imaging
Defining treatment in heartteam
Patient < 65 years
Patient > 65 years
Elderly outpatient clinic
Additional researches if needed
PREDOCS screening: Assessing increased risk of complications after surgery
Pre-operative screening with cardio-thoracic surgeon, anaesthesiologist, E.N.T
- Appointment in 3 weeks with the cardiologist
- Anticoagulation clinic
Waiting for surgery: - Urinary catheterization
- Drugs
- Heart-lung-machine if planned
Time out
Full anesthesia
Endotracheal tube and median sternotomy
Aortic valve replacement
Pacing wires
Sternum and skin is sutured
Start post-operative medication
Patient waking up
- Patient sober from 0.00, until 6.00 a little bit of water
- No own drugs, only pre-drugs at 6.00
- 2 glasses of lemonade before 8.30
- RR, T, pulse, saturation measurements
- No own drugs in the morning, only pre-drugs at 10.00
First patient to OR
Second patient to OR
Ward check-in: - Admission by physician assistant (arts-assistent) - Nursing sensitive measures are filled in - Information about procedure
- ECG
- T, RR, pulse, saturation
- Pre-operative lab
- Length/weight
- Nose culture (if not done yet), urinary culture, urinary sediment
- Nose cream 3 days before
- Anticoagulation
- Echo and X-thorax if needed
- Hair removal
- Patient needs to shower
- Information provision
- Check of aftercare
- Surgeon visits between 4 and 8
- Diabetic patients: daily curve
Recovery:
- RR, T, pulse, saturation
- Urine production
- VAS score 3 times a day
- 72 hours Telemetry
- NaCl
- Removal of drain
- Administration of O2
- Dangle legs
- Physiotherapy
- Physical examination
- Inspection sternum wound, drain plugs, PM wires
- X-Thorax, ECG, lab
- Removal urinary catheter
- Swan lock on drip if needed
- O2 stop
- Mobilizing patiënt
- Removal pacemaker wire
- Removal stitches of drain
Discharge conversation
Emergency
- Independent in Activities of Daily Living
- 24 hours care until 7 days
- Anticoagulation service
- Diet if needed

Supplementary File IV: Work-as-done TAVR and SAVR process maps

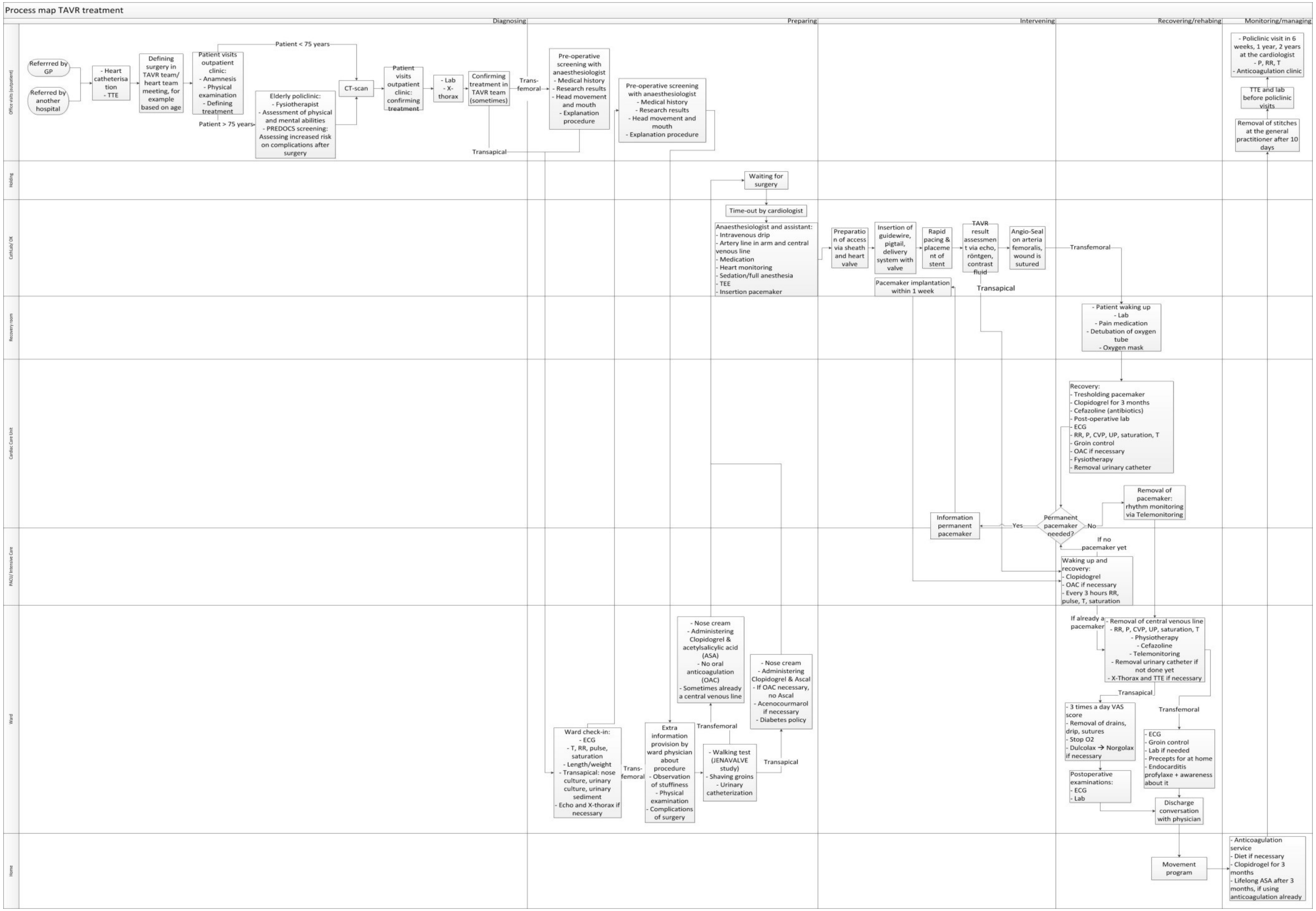

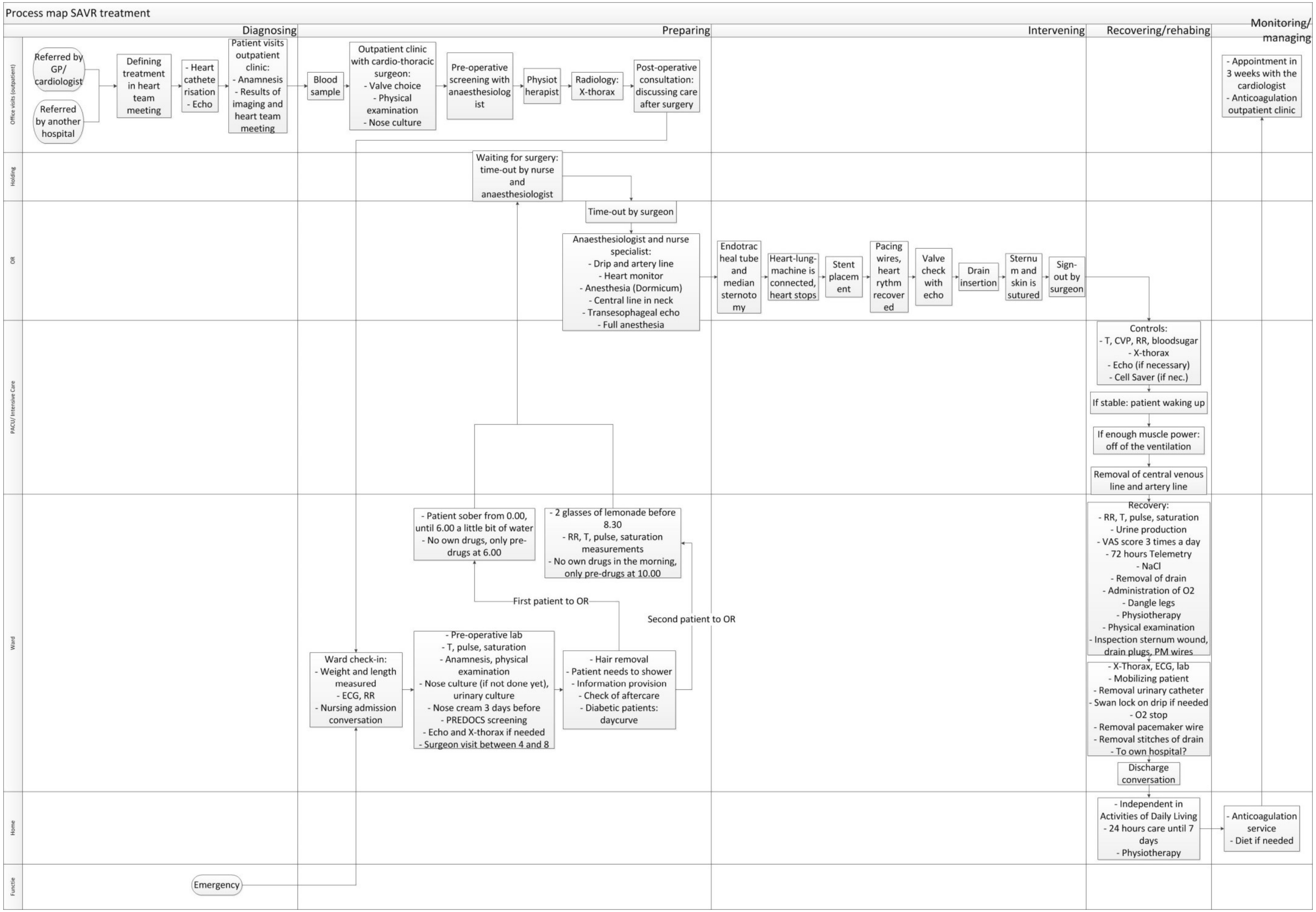
Process map SAVR treatment
Diagnosing
Preparing
Intervening
Recovering/rehabing
Monitoring/ managing
Office visits (outpatient)
Holding
OR
PACU/ Intensive Care
Ward
Home
Functie
Referred by GP/ cardiologist
Referred by another hospital
Defining treatment in heart team meeting
- Heart catheterisation - Echo
Patient visits outpatient clinic: - Anamnesis - Results of imaging and heart team meeting
Blood sample
Outpatient clinic with cardio-thoracic surgeon: - Valve choice - Physical examination - Nose culture
Pre-operative screening with anaesthesiologist
Physiotherapist
Radiology: X-thorax
Post-operative consultation: discussing care after surgery
- Appointment in 3 weeks with the cardiologist - Anticoagulation outpatient clinic
Waiting for surgery: time-out by nurse and anaesthesiologist
Time-out by surgeon
Anaesthesiologist and nurse specialist: - Drip and artery line - Heart monitor - Anesthesia (Dormicum) - Central line in neck - Transesophageal echo - Full anesthesia
Endotracheal tube and median sternotomy
Heart-lung-machine is connected, heart stops
Stent placement
Pacing wires, heart rythm recovered
Valve check with echo
Drain insertion
Sternum and skin is sutured
Sign-out by surgeon
Controls: - T, CVP, RR, bloodsugar - X-thorax - Echo (if necessary) - Cell Saver (if nec.)
If stable: patient waking up
If enough muscle power: off of the ventilation
Removal of central venous line and artery line
Recovery: - RR, T, pulse, saturation - Urine production - VAS score 3 times a day - 72 hours Telemetry - NaCl - Removal of drain - Administration of O2 - Dangle legs - Physiotherapy - Physical examination - Inspection sternum wound, drain plugs, PM wires
- X-Thorax, ECG, lab - Mobilizing patient - Removal urinary catheter - Swan lock on drip if needed - O2 stop - Removal pacemaker wire - Removal stitches of drain - To own hospital?
Discharge conversation
- Patient sober from 0.00, until 6.00 a little bit of water - No own drugs, only pre-drugs at 6.00
- 2 glasses of lemonade before 8.30 - RR, T, pulse, saturation measurements - No own drugs in the morning, only pre-drugs at 10.00
First patient to OR
Second patient to OR
Ward check-in: - Weight and length measured - ECG, RR - Nursing admission conversation
- Pre-operative lab - T, pulse, saturation - Anamnesis, physical examination - Nose culture (if not done yet), urinary culture - Nose cream 3 days before - PREDOCS screening - Echo and X-thorax if needed - Surgeon visit between 4 and 8
- Hair removal - Patient needs to shower - Information provision - Check of aftercare - Diabetic patients: daycurve
- Independent in Activities of Daily Living - 24 hours care until 7 days - Physiotherapy
- Anticoagulation service - Diet if needed
Emergency

Supplementary File V: Substantiation in literature

1. Information provision to patients about SAVR treatment

Our study proposes that a poor timing of mobilization might lead to sternal dehiscence. Important is that sternal dehiscence is not only caused by poor mobilization, but also other risk factors identified by earlier studies, such as obesity.[4] However, using the process measure about 'information provision' might lead to the first steps in the direction of quality improvement of the 'sternal wound infections' result.

2. Valve choice for TAVR and SAVR treatment

We used the process measures of the NHR for TAVR and SAVR prosthesis types in our definition for 'valve choice', which were '*Type of prosthesis of the SAVR*' and '*Type of prosthesis of the TAVR*'.[1] Previous studies concluded that the brand of the valve influences a permanent pacemaker implantation, because CoreValve prostheses showed a higher risk for pacemaker implantation than an Edwards Sapiens prosthesis after TAVR.[5 6] The 'valve choice' might not always be influenceable but measuring the amount of different types of prostheses would give insight in why specific treatment outcomes were found. It could illuminate whether other factors play a role in satisfying or disappointing treatment outcomes.

3. Frailty screening of patients undergoing TAVR and SAVR treatment

Despite the differences in frailty assessment tools between studies, frailty was found to be significantly associated with 1-year and 30-day mortality in multiple earlier studies.[7] No definition or criteria for a frailty score have been given in our study because different criteria are used in the literature.[8 9] Hospitals might want to choose a definition of frailty themselves to use during the elderly outpatients' clinic or the elderly screening but the goal should be the use of an universal frailty score.

It was also recommended in the literature to evaluate the procedural risk of TAVR patients in addition to the decision of the heart team, to prevent that too frail patients are subjected to an inappropriate treatment. In addition to our study, that study proposes to measure the quality of life of TAVR patients before and after the intervention. This is considered important because it indicates the clinical benefit and determines which patients benefit the most of TAVR.[10]

4. Managing waiting lists for TAVR and SAVR treatment

Our process measure 'time between the TAVR surgery indication and surgery' has also been supported in literature. A previous study found that a longer time on the waiting list is associated with higher mortality and morbidity. No threshold period was found below which waiting times were safe because clinical events showed a constant relationship with waiting time.[11] Considering the results of our study, a long waiting list is unfavourable for vulnerable TAVR patients but a too short waiting list for SAVR patients can lead to changes in OR planning and procedural delays, which could lower the quality of life of the patient. The balance between a too short or too long waiting list is of importance here. However, this is subject for further research.

5. Stopping anticoagulants in SAVR treatment

Regarding 'stopping anticoagulation on time', it was confirmed in previous studies that anticoagulation treatment before surgery increases the risk of resternotomy.[12]

6. Permanent pacemaker implantations in TAVR and SAVR treatment

It was found in previous studies that it is debatable whether 'permanent pacemaker implantations' take place fast enough. An association of early pacemaker implantation with death was found, but the permanent pacemaker implantation itself was not leading to lower survival.[13] Moreover, AV conduction disturbances were partially shown to recover over time.[6] Therefore, it is important for a hospital to decide on guidelines regarding the waiting time for heart rhythm to restore.

7. Pain measurement of patients after SAVR treatment

The importance of 'pain treatment' is also emphasized in previous studies because poor pain treatment may lead to for example negative cardiac, pulmonary and musculoskeletal effects. Regular measurement of pain is important in the treatment of pain.[14] We used the Dutch Health and Youth Care Inspectorate's

Akmaz B, *et al. BMJ Open Qual* 2019; 8:e000716. doi: 10.1136/bmjoq-2019-000716

measure to define our process measure for pain management, which was: *'The number of clinical surgical patients whose pain level is recorded digitally at least once a day during each day of admission'*.[15]

References

1. Meetbaar Beter. Handboek Dataverzameling 2017: Meetbaar Beter Foundation 2017:56-68.
2. Registratie NH. Over NHR 2017 [Available from: https://nederlandsehartregistratie.nl/over-nhr/2018.
3. Meetbaar Beter. Meetbaar Beter Boek 2016: Meetbaar Beter Foundation 2016:67.
4. Molina JE, Lew RS, Hyland KJ. Postoperative sternal dehiscence in obese patients: incidence and prevention. *Ann Thorac Surg* 2004;78(3):912-7; discussion 12-7. doi: 10.1016/j.athoracsur.2004.03.038 [published Online First: 2004/09/01]
5. Erkapic D, Kim WK, Weber M, et al. Electrocardiographic and further predictors for permanent pacemaker requirement after transcatheter aortic valve implantation. *EP Europace* 2010;12(8):1188-90. doi: 10.1093/europace/euq094
6. Siontis GCM, Jüni P, Pilgrim T, et al. Predictors of Permanent Pacemaker Implantation in Patients With Severe Aortic Stenosis Undergoing TAVR: A Meta-Analysis. *Journal of the American College of Cardiology* 2014;64(2):129-40. doi: https://doi.org/10.1016/j.jacc.2014.04.033
7. Thongprayoon C, Cheungpasitporn W, Kashani K. The impact of frailty on mortality after transcatheter aortic valve replacement. *Annals of translational medicine* 2017;5(6):144. doi: 10.21037/atm.2017.01.35 [published Online First: 2017/05/04]
8. Rockwood K, Stadnyk K, MacKnight C, et al. A brief clinical instrument to classify frailty in elderly people. *Lancet (London, England)* 1999;353(9148):205-6. doi: 10.1016/s0140-6736(98)04402-x [published Online First: 1999/01/29]
9. Fried LP, Tangen CM, Walston J, et al. Frailty in older adults: evidence for a phenotype. *The journals of gerontology Series A, Biological sciences and medical sciences* 2001;56(3):M146-56. [published Online First: 2001/03/17]
10. Asgar AW, Lauck S, Ko D, et al. Quality of Care for Transcatheter Aortic Valve Implantation: Development of Canadian Cardiovascular Society Quality Indicators. *Can J Cardiol* 2016;32(8):1038.e1-38.e4. doi: http://dx.doi.org/10.1016/j.cjca.2015.11.008
11. Elbaz-Greener G, Masih S, Fang J, et al. Temporal Trends and Clinical Consequences of Wait Times for Transcatheter Aortic Valve Replacement. *Circulation* 2018;138(5):483-93. doi: 10.1161/circulationaha.117.033432 [published Online First: 2018/03/08]
12. Čanádyová J, Zmeko D, Mokráček A. Re-exploration for bleeding or tamponade after cardiac operation. *Interactive cardiovascular and thoracic surgery* 2012;14(6):704-07. doi: 10.1093/icvts/ivs087 [published Online First: 2012/03/20]
13. Greason KL, Lahr BD, Stulak JM, et al. Long-Term Mortality Effect of Early Pacemaker Implantation After Surgical Aortic Valve Replacement. *The Annals of Thoracic Surgery* doi: https://doi.org/10.1016/j.athoracsur.2017.01.083
14. Cogan J. Pain Management After Cardiac Surgery. *Seminars in Cardiothoracic and Vascular Anesthesia* 2010;14(3):201-04. doi: doi:10.1177/1089253210378401
15. Gezondheidszorg Ivd. Basisset Medisch Specialistische Zorg Kwaliteitsindicatoren 2018. In: Ministerie van Volksgezondheid WeS, ed. Utrecht, 2017.
